# Divergent Rhabdovirus Discovered in a Patient with New-Onset Nodding Syndrome

**DOI:** 10.3390/v14020210

**Published:** 2022-01-21

**Authors:** Arthur W. D. Edridge, Gasim Abd-Elfarag, Martin Deijs, Maarten F. Jebbink, Michael Boele van Hensbroek, Lia van der Hoek

**Affiliations:** 1 Laboratory of Experimental Virology, Department of Medical Microbiology and Infection Prevention, Amsterdam UMC, University of Amsterdam, 1105 AZ Amsterdam, The Netherlands; m.deijs@amsterdamumc.nl (M.D.); m.f.jebbink@amsterdamumc.nl (M.F.J.); 2 Center for Global Child Health, Amsterdam UMC, University of Amsterdam, 1105 AZ Amsterdam, The Netherlands; g.o.abdelfarag@amsterdamumc.nl (G.A.-E.); m.boele@amsterdamumc.nl (M.B.v.H.)

**Keywords:** tibroviruses, virus discovery, nodding syndrome, next-generation sequencing, rhabdoviruses

## Abstract

A divergent rhabdovirus was discovered in the bloodstream of a 15-year-old girl with Nodding syndrome from Mundri West County in South Sudan. Nodding syndrome is a progressive degenerative neuropathy of unknown cause affecting thousands of individuals in Sub-Saharan Africa. The index case was previously healthy until she developed head-nodding seizures four months prior to presentation. Virus discovery by VIDISCA-NGS on the patient’s plasma detected multiple sequence reads belonging to a divergent rhabdovirus. The viral load was 3.85 × 10^3^ copies/mL in the patient’s plasma and undetectable in her cerebrospinal fluid. Further genome walking allowed for the characterization of full coding sequences of all the viral proteins (N, P, M, U1, U2, G, U3, and L). We tentatively named the virus “Mundri virus” (MUNV) and classified it as a novel virus species based on the high divergence from other known viruses (all proteins had less than 43% amino acid identity). Phylogenetic analysis revealed that MUNV forms a monophyletic clade with several human-infecting tibroviruses prevalent in Central Africa. A bioinformatic machine-learning algorithm predicted MUNV to be an arbovirus (bagged prediction strength (BPS) of 0.9) transmitted by midges (BPS 0.4) with an artiodactyl host reservoir (BPS 0.9). An association between MUNV infection and Nodding syndrome was evaluated in a case–control study of 72 patients with Nodding syndrome (including the index case) matched to 65 healthy households and 48 community controls. No subject, besides the index case, was positive for MUNV RNA in their plasma. A serological assay detecting MUNV anti-nucleocapsid found, respectively, in 28%, 22%, and 16% of cases, household controls and community controls to be seropositive with no significant differences between cases and either control group. This suggests that MUNV commonly infects children in South Sudan yet may not be causally associated with Nodding syndrome.

## 1. Introduction

Nodding syndrome (NS) is a severe progressive degenerative neuropathy affecting children and young adults in remote areas in Sub-Saharan Africa. NS has largely been neglected, and although thousands of individuals are affected today, the etiology remains unknown, and no preventative or treatment options are available [1]. One hypothesis that has been proposed is that NS may be caused by a novel neurotropic virus [2]. Previous attempts to identify a potential viral cause have focused on a limited number of known viruses and were unsuccessful; however, these studies did not consider novel viruses [3].

To explore a potential novel viral cause of NS, we performed virus-discovery assays using VIDISCA-NGS on the plasma and cerebrospinal fluid of children with new-onset NS. VIDISCA-NGS allows unbiased detection of known and unknown RNA and DNA viruses in a plethora of clinical specimen, and it was previously used to discover viruses such as human coronavirus NL63 [4] and Ntwetwe orthobunyavirus [5]. Here, we report the discovery of a novel rhabdovirus from the plasma of a child with new-onset NS from Mundri West County in South Sudan.

## 2. Materials and Methods

### 2.1. Study Subject Identification, Clinical Assessment, and Sample Collection

The index case and all other study subjects participated in the case–control study of the South Sudan Nodding Syndrome Study project [6]. Cases with NS were identified through screening of households between February 2018 and November 2019 in the Greater Mundri area, the current epicenter of NS in South Sudan. Cases were matched to a healthy household and a community control. Informed consent was obtained from the guardians prior to study inclusion. All subjects were admitted to Lui Hospital as part of the study-related clinical investigation and sample collection. During admission, a detailed medical history was taken, physical examination was performed, and blood and cerebrospinal fluid (cases only) were collected. All samples were frozen at −20 °C for several months and at −80 °C for long-term storage until subsequent analysis. The study protocol was approved by the ethics committee of the Ministry of Health of the Republic of South Sudan (approval date: 9 December 2016) and the University of Antwerp, Belgium (reg.nr: B300201526244).

### 2.2. Virus Discovery

VIDISCA-NGS was performed on 220 μL CSF pooled 1:1 with Universal Transport Medium (Copan, Murrieta, CA, USA) and on 110 µL EDTA plasma as previously described [7]. Briefly, samples were centrifuged and treated with TURBO DNase (Thermo Fisher Scientific, Waltham, MA, USA) to remove cells and chromosomal DNA. Nucleic acids were manually extracted [8], which was then followed by reverse transcription and second-strand synthesis using non-ribosomal hexamers. Double-stranded DNA was digested by MseI (T^TAA; New England Biolabs, Ipswich, MA, USA) and ligated to an adaptor containing a sample identifier sequence. The library was amplified by PCR with a pre- and post-PCR size selection step using AMPure XP beads (Beckman Coulter, BA CA, USA) to retain DNA with a length between 100 and 400 nucleotides. Seventy sample-libraries were pooled at an equimolar concentration, then clonally amplified on beads using the Ion Chef System (Thermo Fisher Scientific, Waltham, MA, USA) and sequenced on the Ion PGM System (Thermo Fisher Scientific) with an ION 316 Chip (Thermo Fisher Scientific). Sequenced reads were analyzed to be of viral origin using three bioinformatic pipelines: an in-house method aligning translated protein sequences to a curated virus database, the VIDISCA bioinformatics workflow [9], and an online metagenomics profiler [10].

### 2.3. Genome Characterization and Analysis

The near-complete viral genome was obtained through a combination of Illumina sequencing [7] and genome walking. Genome walking was performed on regions with low or absent sequence coverage using primers based on known flanking regions or conserved rhabdovirus sequences when no known flanking regions were available. The PCR products were characterized using Sanger sequencing. De novo assembly was performed using CodonCode Aligner (version 6.0.2) and the resulting contig was manually curated. The curated genome was uploaded to GenBank (accession number OM320812) (https://www.ncbi.nlm.nih.gov/genbank) (accessed on 19 January 2021). Open reading frames (ORFs) were identified and translated into amino acid sequences by NCBI ORF finder (https://www.ncbi.nlm.nih.gov/orffinder) (accessed on 19 January 2021). Amino acid sequences from structural proteins were aligned to those from other rhabdovirus RefSeq sequences from GenBank using all available alignment methods on M-Coffee [11]. MEGA7 software version 7.0.21 was used to find the best protein Maximum Likelihood (ML) complete deletion model between all available methods. The model with the lowest Bayesian Information Criterion scores was used to infer the phylogenies with 500 bootstrap replicates. BioEdit software version 7.2.5 was used to create an amino acid identity matrix from the M-Coffee alignment. Signal peptides were predicted using SignalP 5.0 (https://services.healthtech.dtu.dk/service.php?SignalP-5.0) (accessed on 19 January 2021) and transmembrane domains using DeepTMHMM (https://biolib.com/DTU/DeepTMHMM) (accessed on 19 January 2021). Potential glycosylation sites were predicted using NetOGlyc 4.0 (https://services.healthtech.dtu.dk/service.php?NetOGlyc-4.0) (accessed on 19 January 2021) and NetNGlyc 1.0 (https://services.healthtech.dtu.dk/service.php?NetNGlyc-1.0) (accessed on 19 January 2021). A potential vertebrate host and arthropod reservoir was predicted using Viral Host Predictor (https://bioinformatics.cvr.ac.uk/software/viral-host-predictor/) (accessed on 19 January 2021).

### 2.4. Reverse Transcription Quantitative Polymerase Chain Reaction (RT-qPCR)

A forward primer (5′-AGTTAAACCCCAGAGATTGT-3′), reverse primer (5′-CACCGTGTTAAGGTTTACAC-3′) and FAM-labelled probe (5′-AGTCGGGGGTGATGGATCTGGAGGT-3′) were developed on the polymerase gene of the virus. A dilution series of a TOPO TA plasmid clone (Thermo Fisher Scientific) of the PCR amplicon from the index case was used for calibration. The qPCR was performed on a Rotor-Gene (Qiagen, Hilden, Germany) platform with the Rotor-Gene Probe PCR Kit (Qiagen, Hilden, Germany) using 40 cycles and the manufacturer’s settings using cDNA. The cDNA was obtained by reverse transcription using the SuperScript II (Thermo Fisher Scientific) of the extracted nucleic acids from the patient’s plasma and CSF.

### 2.5. Serological Assessment

A synthetic DNA, coding for the nucleocapsid protein of MUNV, was cloned in a pET-100D TOPO vector (Thermo Fisher Scientific) according to the manufacturer’s protocol. Positive colonies were screened by PCR using T7 and T7 reverse primers and confirmed by Sanger sequencing the inserts. Expression of the recombinant full nucleocapsid protein of MUNV was performed by the transformation of 15 ng plasmid in chemically competent E. coli BL-21 STAR [DE3] (Thermo Fisher Scientific). An overnight culture of the transformed bacteria was inoculated into Luria Broth (LB) medium, supplemented with 1% glucose and carbenicillin (10 µg/mL). The culture was grown to exponential phase prior to inoculation with 0.5 mM of Isopropyl β-D-1-thiogalactopyranoside (IPTG) for 5 h. Recombinant proteins were purified with Ni-NTA (Qiagen), and concentration was measured with a Nano-Drop (Thermo Fisher Scientific). ELISA was performed as described [12].

## 3. Results

In August 2018, a 15-year-old girl was evaluated at the NS study clinic at Lui Hospital, Mundri East County, South Sudan, for clinical and laboratory evaluation for study purposes. The patient met the case definition [13] of probable NS with severity stage 2 [14]. She was previously healthy until she developed nodding seizures in April 2018. The nodding seizures were triggered by food and cold weather and were succeeded by generalized convulsions for which she took anticonvulsive therapy. Besides generalized seizures, she also reported weight loss since the onset of NS symptoms. On clinical examination, the patient had an axillary temperature of 36.2 °C, a heart rate of 100 bpm, a respiratory rate of 28 bpm, a blood pressure of 90 over 60 mmHg, and a body mass index (BMI) of 18.4. Physical (including neurological) examinations were normal. On laboratory examination, microscopy analysis revealed presence of *Mansonella perstans* microfilaria in a blood smear. Hemoglobin measurement, analysis of complete blood count, urine dipstick and microscopy on blood, skin snip and cerebrospinal fluid were all normal. An older brother also met the case-definition for probable NS; an older sister was healthy. At 6 and 12 months after the initial examination at Lui Hospital, the patient was brought to the hospital for the study follow-up. At 6 months, the patient reported generalized convulsions and had developed further weight loss (BMI 16.4). At 12 months, the clinical presentation remained unchanged.

**Figure 1:**
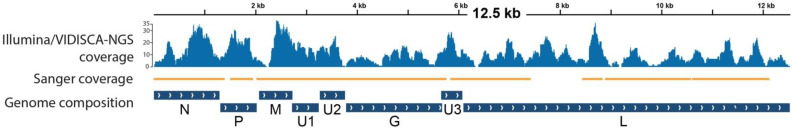
Genome sequence coverage and genome composition of Mundri virus.

Plasma and cerebrospinal fluid were analyzed by VIDISCA-NGS to identify known and unknown viruses. VIDISCA-NGS identified 19,505 sequence reads in the patient’s plasma and 26,233 in her CSF. Several reads from the patient’s plasma belonged to a divergent rhabdovirus which prompted further genomic characterization by Illumina sequencing and genome walking on low-coverage regions. Accordingly, the complete coding sequence of the genome could be characterized (Figure 1). An RT-qPCR developed for the virus identified a viral load in the patient’s plasma of 3.85 × 10^3^ RNA copies/mL. No viral reads could be detected in the CSF VIDISCA-NGS library, nor could viral RNA be detected in the patient’s CSF by RT-qPCR; the viral load was undetectable in her plasma at the 12-month follow-up.

**Figure 2:**
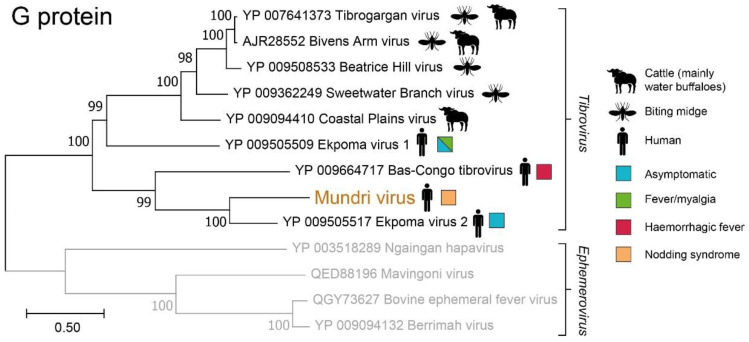
Phylogenetic reconstruction of Mundri virus G protein with its closest ancestors.

The virus genome encodes for eight viral proteins: a nucleocapsid (N), a phosphoprotein (P), a matrix protein (M), a glycoprotein (G), a large protein (L, RNA-dependent polymerase), and three accessory proteins (U1, U2, and U3, Figure 1 and Table 1). Phylogenetic analysis on the amino acid sequences from the structural proteins showed that the virus consistently shares a closest common ancestor with Ekpoma virus 2 (EKV2) and belongs to a monophyletic clade of tibroviruses (family: *Rhabdoviridae*, genus: *Tibrovirus*, Figure 2 and Supplementary Figure S1). The genome composition, protein length and protein structure were like EKV2 (Table 1) and other known tibroviruses [15]. Yet, because the N and the G protein sequences share at most 57% amino acid identity with its closest relatives (Table 2), we propose this virus to be a novel virus species, in line with ICTV criteria [16]. The virus was named Mundri virus (MUNV), after the area where the patient came from.

**Figure 3:**
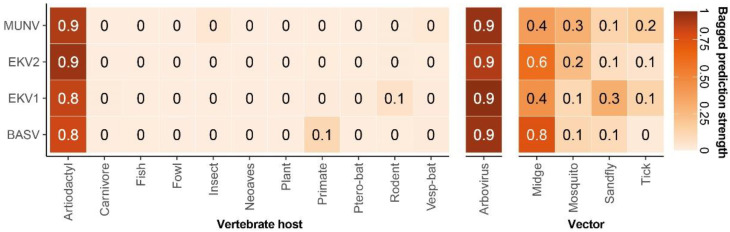
Host prediction of human-infecting tibroviruses using a machine-learning algorithm.

The MUNV genome was analyzed by a machine-learning algorithm to predict its potential vertebrate host and arthropod vector [17]. This algorithm suggested MUNV to be an arbovirus (bagged prediction score of 0.9 (BPS)) transmitted by midges (BPS 0.4) with an artiodactyl (even-toed ungulates) host reservoir (BPS 0.9, Figure 3).

To evaluate whether MUNV may be causally associated with NS, we tested the sample of a case–control study consisting of 72 NS cases (including the index case), 65 matched household controls (including a healthy sibling of the index case), and 48 matched community controls. Using an RT-qPCR to detect MUNV RNA, all samples remained negative except for the index case.

**Figure 4:**
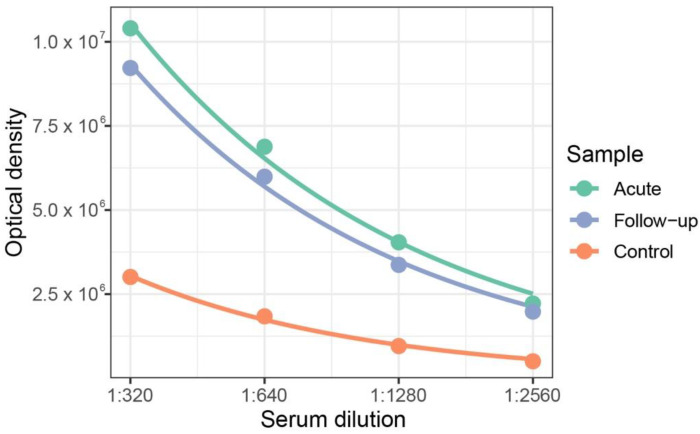
ELISA dilution series specific for Mundri virus anti-nucleocapsid IgG antibodies of index case acute, index case 12-month follow-up, and Dutch control sample.

**Figure 5:**
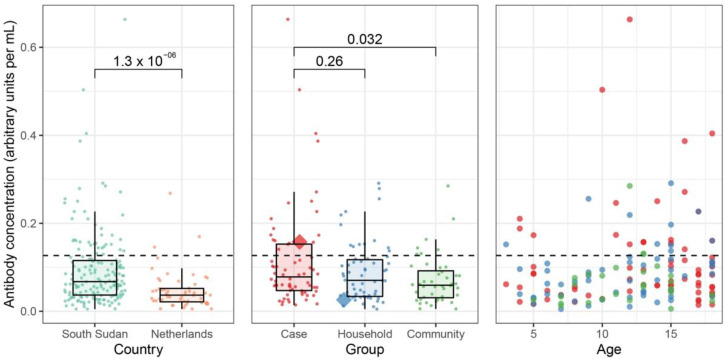
(**Left panel**) Mundri virus (MUNV) anti-nucleocapsid IgG concentrations in South Sudanese children and Dutch controls. (**Middle panel**) MUNV anti-nucleocapsid IgG concentrations amongst NS cases, household controls and community controls. Large red diamond: index case, large blue diamond: healthy sibling of the index case. (**Right panel**) MUNV anti-nucleocapsid IgG concentrations across different age groups in South Sudanese children: cases (red), household controls (blue), and community controls (green). The dotted line represents the seropositivity cut-off: 0.127 AU/mL, based on 2 standard deviations above the median antibody concentration of the Dutch controls. Differences between groups were calculated using the Mann–Whitney U test.

We also developed a serological assay detecting anti-nucleocapsid IgG antibodies. The index case had high levels of MUNV antibodies at presentation compared to a Dutch control sample, which marginally decreased at the 12-month follow-up (Figure 4). To define a seropositivity threshold, we tested a larger cohort of negative controls (Dutch anonymized serum samples), assuming MUNV does not circulate in the Netherlands. Indeed, samples collected from the South Sudanese population contained nearly a two-fold higher median antibody concentration than the samples from the Dutch controls (0.0675 vs. 0.0368 AU/mL, respectively, *p* = 1.3 × 10^−6^, Mann–Whitney U test). Accordingly, a seropositivity threshold was defined at two standard deviations above the Dutch median antibody concentration: 0.127 AU/mL (Figure 5, left panel), above which nearly all Dutch samples (52 of 55) remained seronegative. Applying this threshold on the case–control study, we found 22% (39 of 172) of the South Sudanese population to be seropositive. Interestingly, while the index case was seropositive, her healthy sibling remained seronegative (Figure 5, middle panel). Nonetheless, we found no significant differences in the overall seroprevalence between the NS cases and the household controls (28% vs. 22%, respectively, *p* = 0.42, Mann–Whitey U test), nor between the NS cases and the community controls (28% vs. 16%, *p* = 0.17, Mann–Whitney U test). Comparing absolute antibody concentrations, we found a marginally significant higher concentration of antibodies in the NS cases than in the community controls (0.078 versus 0.059 AU/mL, respectively, *p* = 0.032, Mann–Whitney U test), yet no difference was found between the NS cases and the household controls (0.078 versus 0.070 AU/mL, respectively, *p* = 0.260, Mann–Whitney U test).

Age was not significantly associated with antibody concentration (*p* = 0.13, linear regression, Figure 5, right panel) nor with seropositivity (*p* = 0.33, logistic regression) amongst the South Sudanese children. There was no clustering of seropositive samples amongst children living in the same household (cases and household controls, Supplementary Table S1, *p* = 1, Fisher’s exact test). Lastly, there was no association between *M. perstans* microscopical positivity and MUNV seropositivity (Supplementary Table S2, *p* = 0.12, Fisher’s exact test).

## 4. Discussion

Mundri virus (MUNV) is a divergent rhabdovirus discovered in the bloodstream of a child with new-onset Nodding syndrome. Rhabdoviruses are negative-sense RNA viruses known to infect a wide range of plants, vertebrates, and invertebrates. Only few rhabdoviruses infect humans, but when they do, they may cause severe central nervous system (CNS) infection, such as rabies virus and Chandipura virus [18]. Within the family *Rhabdoviridae*, MUNV seems to be a novel species of genus *Tibrovirus*. Tibroviruses are known to circulate in humans, artiodactyls (even-toed hoofed animals) and *Culicoides* (biting midges, Figure 2). Three human-infecting tibroviruses were known previously, all recently discovered in Africa: Ekpoma virus 1 (EKV1), Ekpoma virus 2 (EKV2), and Bas-Congo virus (BASV). EKV1 and EKV2 were both discovered in the blood of healthy Nigerian women [19]. While EKV1 has not been detected again, EKV2 was later found in the blood of a Chinese laborer in Angola with self-limiting yellow fever-like symptoms [15]. BASV was discovered in the blood of a Congolese nurse and suggested to be the cause of haemorrhagic fever in the nurse and two children [20]. However, this causal link was later disputed [15,21]. Seroprevalence studies on EKV2 have shown that infections may be common; 45% of people living around Ekpoma, Nigeria (the village where the index case lived) were seropositive [19].

Since MUNV belongs to *Rhabdoviridae*—a family of viruses known to cause CNS infections in humans—and was detected in a child with an encephalopathy of unknown cause, we evaluated a potential causal association. We determined whether infections with MUNV were more common in patients with NS than healthy children living in the same household (household controls) or living in the same community (community controls). Using RT-qPCR, no additional MUNV RNA positive cases or controls could be detected besides the index case. Moreover, we were unable to detect MUNV RNA in the CSF of the index case, arguing against a CNS infection. Next, as MUNV may only have caused a temporary infection, or may no longer have been detectable in the bloodstream at the time of sample collection, we also investigated evidence of prior infection by serology. No significant difference in seroprevalence was found between the NS cases and the household controls, nor between the NS cases and the community controls. While we cannot exclude cross-reactivity with other rhabdoviruses—as seen between EKV1 and the rabies virus [19]—our results do not support a direct or indirect causal association between MUNV infection and NS. Instead, given the high overall seroprevalence in the overall South Sudanese cohort (22%), it seems that MUNV commonly infects children in Mundri, South Sudan and generally causes mild disease or asymptomatic infection.

A machine-learning algorithm predicted MUNV to be an arbovirus transmitted by midges with an artiodactyl host reservoir. Midges are also known to transmit many other pathogens, including filaria such as *Mansonella perstans*, for which the index case was also positive. This co-infection of pathogens with a common vector suggests that individuals exposed to one are at increased risk of infection by the other. Although we found no association between MUNV seropositivity and *Mansonella perstans* infections, this may be a result of temporality and still be true for acute infections. Interestingly, several studies have found an association between NS and filarial infection (both with *Onchocerca volvulus* and *Mansonella perstans*), although proof of causality is still lacking [1]. Consequently, if indeed a filarial infection causes NS, this may explain the small differences in MUNV seroprevalence and antibody concentration observed in this study.

The discovery of MUNV in the bloodstream of a human, which was not predicted to be a host reservoir, suggests that humans are accidental and potential dead-end hosts [15,17]. Given that multiple other tibroviruses have been detected in cattle (mainly water buffaloes, Figure 2), future research attempting to establish the host reservoir of MUNV and other human-infecting tibroviruses may focus on these animals first.

The ability to detect novel viruses has greatly improved with the decreased costs of metagenomic next-generation sequencing. It is estimated that more than half a million yet-to-be-discovered viruses with zoonotic potential are circulating in mammals and birds. This argument has been used to support the Global Virome Project, aiming to characterize these viruses before they become zoonotic [22]. However, such undertakings have been difficult and expensive, and BASV was one of the few human viruses discovered by such projects [23]. Even if all viruses are characterized, it remains questionable whether the risk for zoonotic and epidemic potential can be estimated with sufficient accuracy, or in time to allow effective public health intervention. In the meantime, we advocate for improved early detection of viral threats once they emerge in a human population. This can be achieved by focusing on improved disease surveillance to monitor for outbreaks of unexplained diseases combined with targeted virus-discovery efforts to evaluate a potential novel viral etiology.

## Figures and Tables

**Table 1:** Genome composition of Mundri virus and its closest ancestor, Ekpoma virus 2.

ORF/Protein	Virus	ORF nt Location	Aa Length	Signal Peptide (aa Cleavage Site)	Transmembrane Domain (aa Location)	Glycosylation Sites	
						N	O
1/N	MUNV	1–1284	427	None	None	1	4
	EKV2	48–1331	427	None	None	4	3
2/P	MUNV	1309–2013	234	None	None	2	11
	EKV2	1396–2049	217	None	None	4	17
3/M	MUNV	2058–2705	215	None	None	0	7
	EKV2	2024–2713	229	None	None	2	8
4/U1	MUNV	2702–3226	174	None	None	0	0
	EKV2	2710–3237	175	None	None	0	2
5/U2	MUNV	3245–3736	163	None	None	2	0
	EKV2	3234–3740	168	None	None	1	2
6/G	MUNV	3761–5635	624	1 (18–19)	1 (570–592)	7	6
	EKV2	3950–5842	630	1 (23–24)	1 (577–599)	4	3
7/U3	MUNV	5613–6026	137	None	1 (36–62)	0	3
	EKV2	5844–6221	125	None	1 (23–40)	1	4
8/L	MUNV	6049–12,429	2126	None	None	10	19
	EKV2	6263–12,625	2120	None	None	6	6

ORF: open reading frame, nt: nucleotide, aa: amino acid.

**Table 2:** Amino acid identity matrix of the L and N proteins of Mundri virus with known tibroviruses.

	MUNV	EKV2	EKV1	BASV	TIBV	BAV	BHV	SWBV	CPV
MUNV	ID	0.568	0.414	0.45	0.415	0.416	0.415	0.411	0.415
EKV2	0.570	ID	0.409	0.468	0.407	0.407	0.406	0.426	0.413
EKV1	0.409	0.411	ID	0.423	0.414	0.414	0.414	0.421	0.438
BASV	0.432	0.43	0.38	ID	0.396	0.397	0.394	0.397	0.389
TIBV	0.402	0.393	0.473	0.375	ID	0.972	0.862	0.632	0.549
BAV	0.404	0.393	0.475	0.378	0.99	ID	0.857	0.628	0.549
BHV	0.395	0.388	0.466	0.366	0.943	0.946	ID	0.635	0.54
SWBV	0.388	0.379	0.454	0.368	0.752	0.759	0.747	ID	0.546
CPV	0.383	0.395	0.463	0.38	0.665	0.67	0.665	0.677	ID

MUNV: Mundri virus, EKV2: Ekpoma virus 2, EKV1: Ekpoma virus 1, BASV: Bas-Congo virus, TIBV, Tibrogargan virus, BAV, Bivens Arm virus, BHV Beatrice Hill virus, SWBV: Sweetwater Branch virus, CPV: Coastal Plains virus. Blue: L protein, green: N protein.

## Data Availability

DNA and amino acid sequences from the MUNV genome will be uploaded to GenBank upon publication of this manuscript.

## Supplementary Materials

The following are available online at https://www.mdpi.com/article/10.3390/v14020210/s1, Figure S1: Phylogenetic reconstruction of Mundri virus L, N, M and P proteins with its closest relatives. Table S1. No clustering of Mundri virus seropositivity among children living in the same household in South Sudan. Table S2. No association between Mundri virus seropositivity and *M. perstans* microscopical positivity.
