# Supplementary material for: Divergent Rhabdovirus Discovered in a Patient with New-Onset Nodding Syndrome
Source: Viruses. 2022 Jan 21;14(2):210. doi: 10.3390/v14020210 (PMC8880091; doi:10.3390/v14020210)
Supplement: Supplementary file 1 [file viruses-14-00210-s001.zip › viruses-1474099-supplementary.pdf]

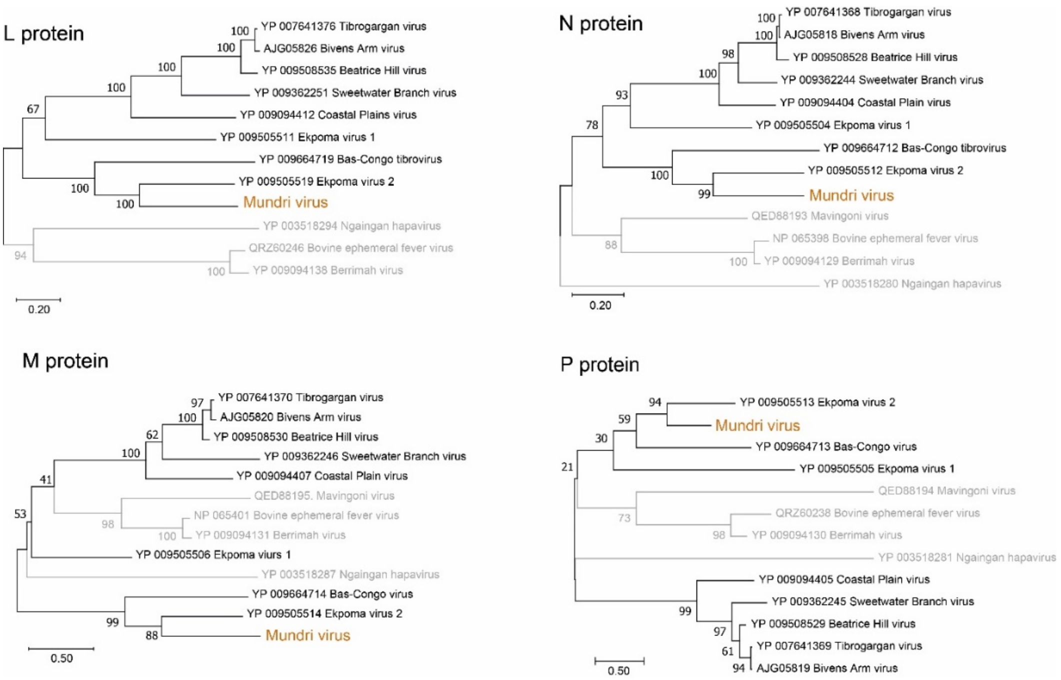


**Figure S1.** Phylogenetic reconstruction of Mundri virus L, N, M and P proteins with its closest ancestors.

**Table S1.** No clustering of Mundri virus seropositivity among children living in the same household in South Sudan.

| | | Household Controls | | |
|---|---|---|---|---|
| | | **Negative** | **Positive** | **Total** |
| **NS cases** | **Negative** | 33 | 9 | 42 |
| | **Positive** | 13 | 4 | 17 |
| | **Total** | 46 | 13 | 59 |

$p$ value = 1, Fisher's exact test.

**Table S2.** No association between Mundri virus seropositivity and *M. perstans* microscopical positivity.

| | | Mundri Virus | | |
|---|---|---|---|---|
| | | **Seronegative** | **Seropositive** | **Total** |
| ***M. perstans*** | **Negative** | 82 | 27 | 109 |
| | **Positive** | 35 | 5 | 40 |
| | **Total** | 117 | 32 | 149 |

$p$ value = 0.12, Fisher's exact test.
